# Roles of skeletal muscle-derived exosomes in osteoporosis

**DOI:** 10.1186/s12967-025-07315-3

**Published:** 2025-11-18

**Authors:** Yuting He, Yuxuan Wang, Kaihong Weng, Xiquan Weng, Yu Yuan

**Affiliations:** 1 School of Exercise and Health, Guangzhou Sport University, Guangzhou, 510500 China; 2 Guangdong Provincial Key Laboratory of Physical Activity and Health Promotion, Guangzhou Sport University, Guangzhou, 510500 China

**Keywords:** Skeletal muscle-derived exosomes, Osteoporosis, Cross-organ regulation, Bone metabolism, Muscle-bone crosstalk

## Abstract

Bone maintains a relatively stable bone mass by balancing bone formation and resorption. The development of osteoporosis is closely associated with the disruption of this balance. Muscles and bones, integral components of the musculoskeletal system, are functionally interconnected, and the onset of osteoporosis is frequently linked to the decline in skeletal muscle function. Exosomes play a crucial role in facilitating chemical information exchange between muscles and bones. This study aims to elucidate the effects of skeletal muscle-derived exosomes on bone formation and resorption, investigate their therapeutic potential for osteoporosis, and propose novel strategies for osteoporosis treatment and targeted drug development. The Translational Potential of this Article: This study investigated the potential of skeletal muscle-derived exosomes in osteoporosis treatment, elucidating their critical role in modulating bone formation and resorption. By clarifying the interaction mechanisms between muscle and bone mediated by exosomes, this research laid a theoretical foundation for novel therapeutic strategies. Therapies based on serve as more targeted and efficient interventions with fewer side effects, thereby advancing the field of bone tissue engineering and offering new prospects for the prevention and management of osteoporosis.

## Introduction

Osteoporosis is a chronic disease arising from the disruption of homeostasis between bone formation and resorption [1]. It is commonly associated with aging and hormonal changes, characterized by an accelerated bone loss rate, bone microstructure degradation, reduced BMD(bone mineral density), and increased bone fragility. These changes significantly elevate the risk of fractures in middle-aged and elderly individuals and severely impair their quality of life [2]. However, current pharmacological interventions, such as bisphosphonates and Teriparatide, are limited by toxic side effects and have not fundamentally addressed the underlying causes of osteoporosis [3]. Therefore, there is an urgent need to explore novel therapeutic strategies for managing osteoporosis.

Skeletal muscle, as an organ closely attached to bone, shares a functional interdependence with the skeletal system. Recent studies indicate that the development of osteoporosis is closely linked to the decline in skeletal muscle function [4]. Appropriate exercise has been shown to enhance muscle mass, effectively increase bone mass, and alleviate osteoporosis symptoms [5, 6]. Furthermore, accumulating evidence suggests that the interaction between muscles and bones extends beyond mechanical stress. Myokines, such as irisin and interleukins, secreted by skeletal muscle, play critical roles in regulating bone metabolism [7]. For instance, exercise can modulate the expression of interleukin-15 derived from skeletal muscle cells, indirectly influencing both bone and muscle functions [8]. Additionally, recent studies reveal that skeletal muscle can regulate bone metabolism through the secretion of exosomes [9]. Exosomes, acting as key mediators of cross-organ communication, are widely secreted by various cell types [10]. Under different physiological conditions, skeletal muscle secretes varying amounts of exosomes, which carry diverse signaling molecules, including proteins and nucleic acids, to regulate bone metabolism [11]. Moreover, the unique ability of exosomes to encapsulate and deliver signaling molecules offers new avenues for treating various diseases, particularly those involving organs with close functional connections, such as bone and muscle [12, 13]. This review explores the potential applications of skeletal muscle-derived exosomes in the field of bone tissue engineering, and particularly emphasizes the regulatory roles of exosomes in osteoporosis, providing a theoretical basis for the treatment of osteoporosis as well as the research on its mechanism.

### The biological functions of exosomes

**Fig. 1:**
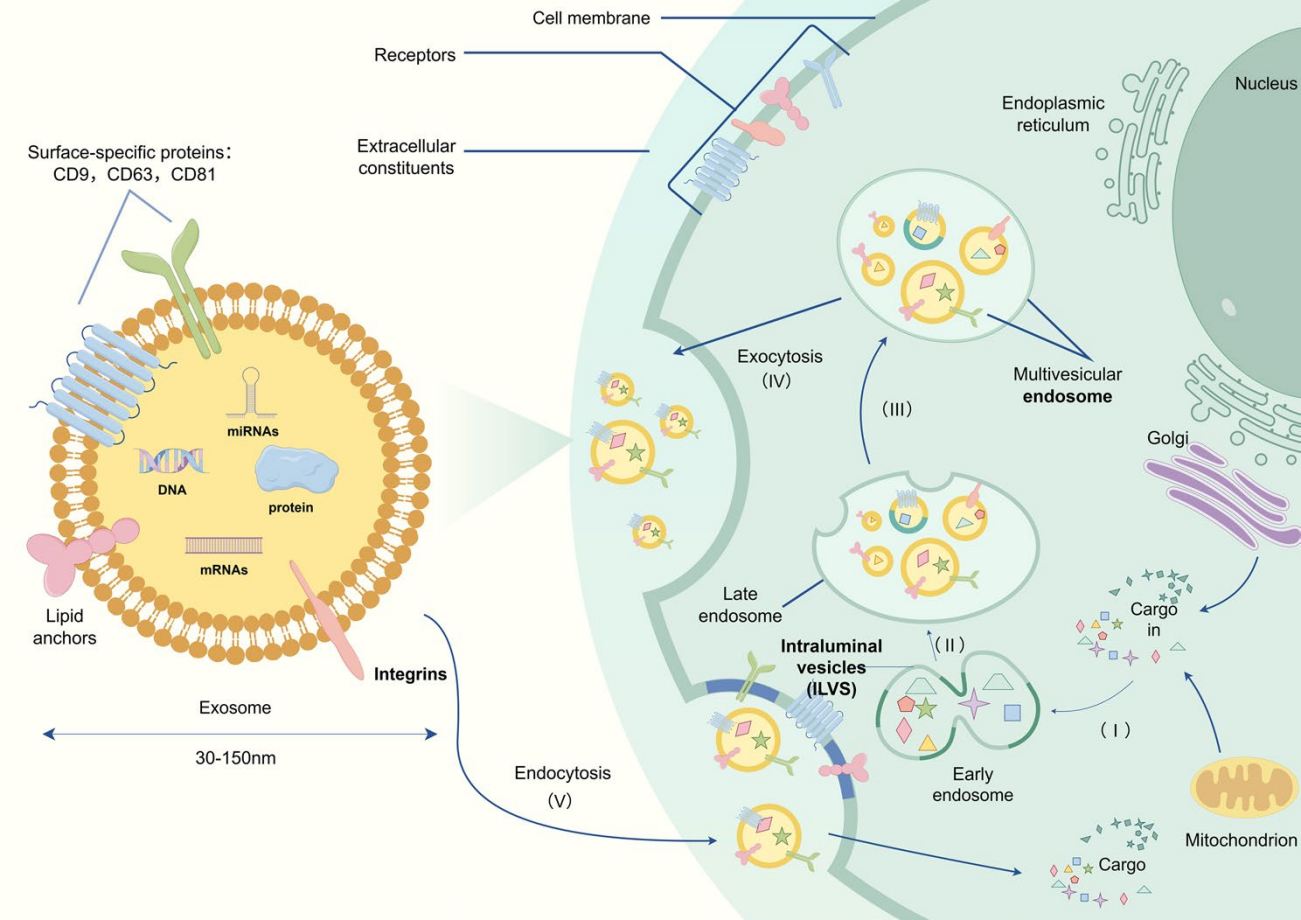
Structure and biological functions of exosomes. Exosomes originate from early endosomes (**I**) within the cell, progressing through multivesicular bodies (**II**) and late endosomes (**III**), and are ultimately secreted into the extracellular space via exocytosis (**IV**). Exosomes carry a variety of components, including dna, miRNAs, mRNAs, and proteins, and exhibit specific surface markers such as CD9, CD63, and CD81. By binding to receptors on the cell membrane, exosomes mediate intercellular signaling and communication. The arrows depict the biogenesis and secretion pathways of exosomes, while lipid anchors and integrins contribute significantly to their stability and targeting specificity

Exosomes are a specific subtype of extracellular vesicles (EVs) derived from the endocytic pathway, with a size range of 30–150 nm and enriched in markers such as CD9, CD63, CD81, Alix, HSP70, and TSG101 [14]. EVs, a heterogeneous group of membrane-bound vesicles, can be divided into microvesicles, apoptotic vesicles (ApoVs), and exosomes. Microvesicles (50–1000 nm), formed by direct outward budding of the plasma membrane, are marked by Annexin A1 and ARF6. They play critical roles in tumor metastasis, coagulation, and inflammatory responses [15]; ApoVs (100–5000 nm), generated upon plasma membrane rupture during apoptosis and identified by Annexin V and histone H3, play a role in clearing apoptotic debris. [16]. Exosomes are formed by the budding of endosomal membranes to generate intraluminal vesicles (ILVs) and multivesicular endosomes (MVEs), which are then released through the plasma membrane [17]. RNA, DNA, lipids, and proteins selectively enter exosomes during their formation as intraluminal vesicles [18] (Fig. 1). Previous studies have demonstrated that exosomes are secreted by various tissues and organs, including skeletal muscle [19], heart [20], liver [21], kidney [22], and adipose tissue [23]. They can act on secreting tissues and neighboring tissues via endocytosis or be transported to distant tissues and organs throughout the body via the bloodstream [24, 25]. Currently, methods such as ultracentrifugation, size exclusion chromatography, ultrafiltration, and IAC (immune affinity capture) enable the effective extraction of exosomes from cells and tissues [26–28]. Additionally, techniques for identifying exosomes, including NTA(nanoparticle tracking analysis), flow cytometry, electron microscopy, and marker protein identification, have become relatively mature [29–32]. Due to their content of genetic information-carrying biomolecules, exosomes facilitate cellular regulation under normal physiological conditions. A typical example is the ability of mesenchymal stem cells to stimulate cell growth and differentiation via exosome-mediated signaling. For instance, bone marrow mesenchymal stem cells promote bone and bone vascular formation by transmitting long non-coding RNAs through exosomes [33, 34]. Under pathological conditions, disease-induced changes in secreting tissues alter exosome quantity, morphology, distribution, and cargo, leading to differences compared to healthy tissues. Consequently, exosomes serve as effective biomarkers for diagnosing and predicting certain diseases [35, 36]. For example, elevated levels of tsRNA(tRNA-derived small noncoding RNA) in urinary exosomes of lupus nephritis patients exhibit high sensitivity and specificity [37]. Furthermore, the layered lipid structure of exosomes confers biocompatibility and reduces toxicity and immunogenicity, making them promising carriers for drug delivery distinct from traditional approaches [38]. However, challenges remain in improving drug loading efficiency, targeting specificity, and scalability of separation and purification processes [39]. Therefore, exosomes have emerged as research focal points in cross-organ communication, disease biomarker discovery, and innovative drug delivery strategies [40].

### The influence of exosomes on bone metabolism

Upon release from cells via budding, exosomes exert paracrine effects on neighboring tissues or are transported systemically through the bloodstream to regulate distant tissues. Consequently, almost all tissues both secrete and absorb exosomes, influencing their own metabolic processes [41]. In some studies, it is not clearly specified whether the extracellular vesicles examined are exosomes [17]. Therefore, in this review, extracellular vesicles with a particle size of less than 150 nanometers, as determined by NTA(nanoparticle tracking analysis), and that express exosome-specific marker proteins are classified as exosomes [41].

A large number of studies have demonstrated that exosomes from various sources influence bone metabolism. Among these, exosomes derived from BMSCs(bone marrow mesenchymal stem cells) play a significant role in regulating bone metabolism [42]. BMSCs are multipotent cells in the bone marrow and can differentiate into bone, cartilage, fat, and other tissues [43–46]. For instance, miR-25 derived from BMSCs can regulate the ubiquitination of Runx2(Runt-Related Transcription Factor 2) via Smurf1(SMAD Specific E3 Ubiquitin Protein Ligase 1), thereby participating in bone metabolic processes [47]. MicroRNAs (miRNAs) are small endogenous non-coding RNAs capable of modulating the expression of protein-coding genes [48], and extracellular vesicles serve as key carriers for miRNA-mediated intercellular communication [49]. In recent years, an increasing body of evidence has shown that miRNAs carried by BMSC-derived exosomes play crucial roles in the treatment of bone-related diseases: Specifically, miR-150-3p [50], miR-27a-3p, and miR-196b-5p [51] derived from BMSC exosomes promote osteogenic differentiation and positively regulate bone remodeling. Additionally, miR-21-5p regulates KLF3 (Kruppel-like factor 3) to promote osteoblast proliferation, contributing to the improvement of osteoporosis [52]. The specific transfer of BMSC-derived exosomes from normal mice to diabetic mice enhances bone mass and reduces fat accumulation in the bone marrow [53]. Furthermore, upregulation of miR-140-3p in BMSC-derived exosomes promotes the differentiation of BMSCs into osteoblasts, accelerates bone regeneration, and alleviates diabetes-induced bone loss [54]. Other components within BMSC exosomes, such as circRNAs(circular RNAs), also influence bone metabolism. For example, circHIPK3, a circular non-coding RNA, targets miR-29a-5p and PINK1 to regulate mitochondrial autophagy and enhance the osteogenic differentiation of MC3T3–E1 cells [55].

**Fig. 2:**
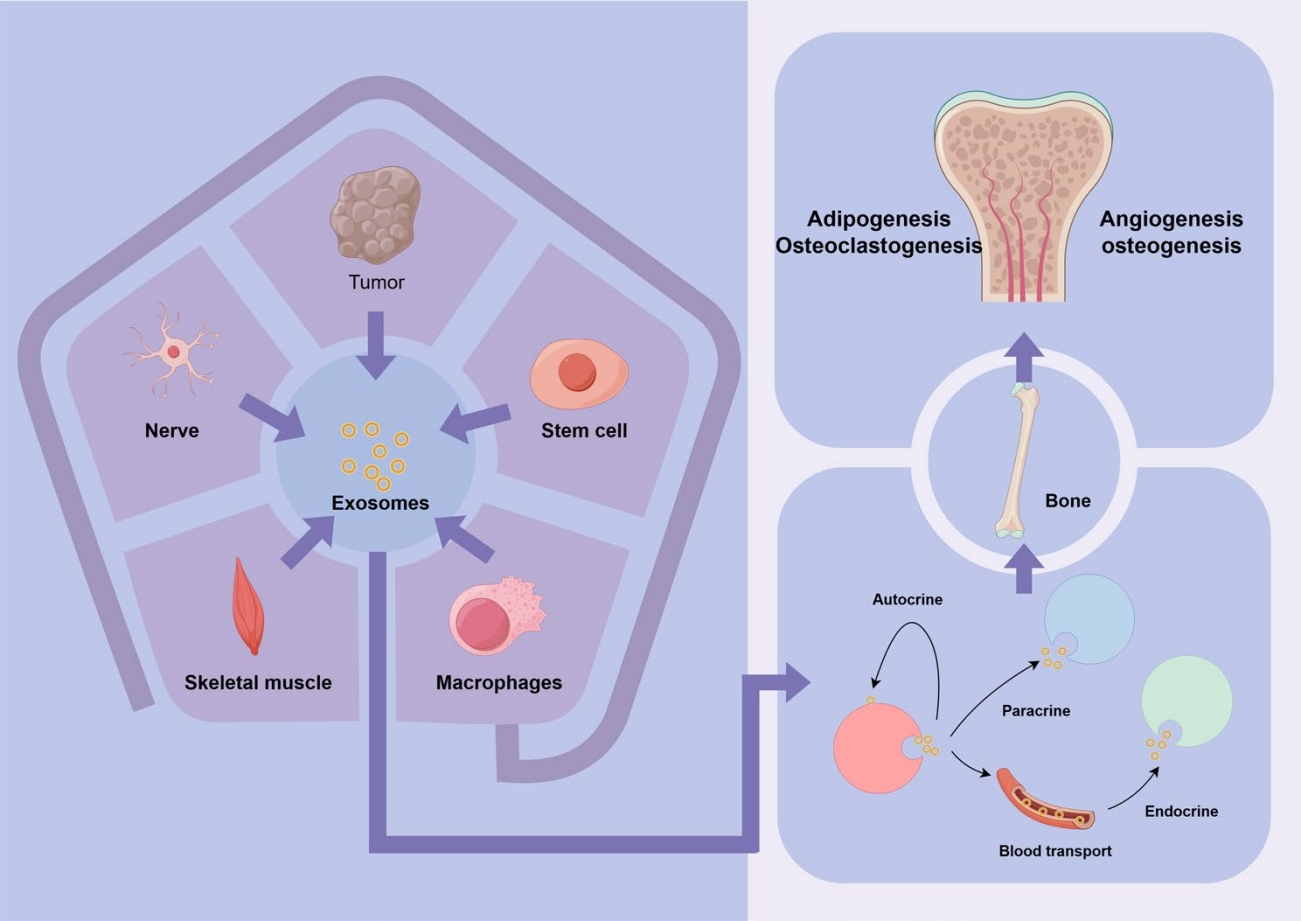
Effects of exosomes from different sources on bone metabolism. Exosomes mediate intercellular communication by transmitting signals between tumors, nerves, skeletal muscles, stem cells, and macrophages. Through systemic circulation via the bloodstream, exosomes modulate the endocrine system through autocrine, paracrine, and endocrine pathways, ultimately influencing angiogenesis, osteogenesis, adipogenesis, and osteoclastogenesis in the bone microenvironment

Apart from BMSCs, various tissues can regulate bone metabolism through exosomes (Fig. 2). For example, exosomes derived from ADSCs(adipose-derived stem cells) enhance angiogenesis and osteogenic effects [56, 57]. ADSC-exos can activate the Wnt/β-catenin signaling pathway to promote the osteogenic differentiation of BMSCs [58], and they also alleviate diabetic osteoporosis by inhibiting osteoclast activity [59]. Exosomes secreted by vascular ECs(endothelial cells) play a critical role in maintaining bone homeostasis [60]. Exosomes derived from HUVECs(human umbilical vein endothelial cells) promote bone formation by inducing M2 polarization of macrophages, while those derived from EPCs(endothelial progenitor cells) enhance bone angiogenesis and accelerate osteogenesis [61]. In addition, the nervous system can also regulate bone metabolism by modulating exosome secretion. Sympathetic stress activates miR-21 transcription in osteoblasts, and miR-21 is transferred to osteoclast progenitor cells via exosomes to inhibit osteoclastogenesis [62].

As critical mediators of intercellular communication, exosomes play a significant role in regulating diseases such as osteoporosis. Given the close anatomical and physiological links between muscle and bone, coupled with their reciprocal pathophysiological modulations in musculoskeletal diseases, studying the effects of skeletal muscle-derived exosomes holds dual significance: it promises to decipher fundamental molecular crosstalk mechanisms governing musculoskeletal homeostasis while concurrently establishing a scientific framework for developing exosome-mediated therapeutic interventions targeting osteoporosis.

## Effects of skeletal muscle-derived exosomes on bone metabolism

### Effects of skeletal muscle-derived exosomes on bone formation

The locomotor system comprises bones, skeletal muscles, and joints [63]. As integral components of this system, muscles and bones exhibit a closely interconnected functional relationship. In particular, the mechanical stress generated by muscle contraction during exercise or physical activity significantly influences bone health. Skeletal muscles can modulate the structure of bone tissue through the application of force [64]. Skeletal muscle mainly comprises quiescent satellite cells, differentiating myoblasts, and mature myofibers [65]. Each subpopulation secretes exosomes that differ in particle size, surface markers, and miRNA cargo, thereby exerting distinct effects on bone metabolism [66]. For example, treatment with satellite cell-derived exosomes suppressed TGF-β1/Smad3 signaling, thereby alleviating muscle wasting and fibrosis [67], and thus mitigating bone loss induced by skeletal muscle atrophy [68]. C2C12 myoblast-derived exosomes promote the osteogenic differentiation of pre-osteoblastic cells by activating the β-catenin signaling pathway via miR-27a-3p [69]. Exosomes released by mature myofibers (myotubes) are enriched in miR-27a-3p and miR-486-5p. These microRNAs activate key signaling axes—β-catenin, PTEN/Akt/mTOR, and TGF-β/Smad2/3—thereby markedly promoting osteogenic differentiation and mineralized matrix formation in BMSCs and pre-osteoblastic MC3T3–E1 cells [70].

Furthermore, musculoskeletal disorders often co-occur in aging or chronic diseases. For instance, sarcopenia frequently accompanies osteoporosis during the aging process, reflecting the interdependence of muscle and bone health [71]. This phenomenon arises because skeletal muscles not only regulate bone metabolism via biochemical pathways but also influence bone homeostasis through changes in mechanical signals. For example, irisin secreted by skeletal muscle inhibits osteoclast formation and bone resorption [72]. Additionally, irisin induces mitochondrial autophagy in osteoblasts and enhances the phosphorylation of AMPK(AMP-Activated Protein Kinase) and ULK1(Unc-51 Like Autophagy Activating Kinase 1), thereby mitigating bone loss [73]. Meanwhile, myostatin, a myokine secreted by skeletal muscles, directly affects osteoblast function and indirectly suppresses osteoblast differentiation by downregulating exosomal miR-218 derived from osteocytes, thus influencing bone metabolism [74]. Skeletal muscle-derived exosomes serve as critical carriers of signaling molecules that mediate cross-organ communication between muscles and bones.

The regulatory role of skeletal muscle-derived exosomes in bone metabolism has been validated in mice. In a study where healthy mouse skeletal muscle EVs(extracellular vesicles) were injected via the tail vein into mice with myopathy and osteoporosis, it was observed that while these EVs did not mitigate muscle loss, Micro-CT analysis revealed significant improvements in BMD(bone mineral density), BV/TV(bone volume to tissue volume ratio), cortical bone thickness, and trabecular bone structure in osteoporotic mice. These findings indicate enhanced bone mass and trabecular architecture. Additionally, Trap(Tartrate-Resistant Acid Phosphatase) staining of femurs demonstrated that the number of osteoclasts in osteoporotic mice treated with normal mouse EVs closely resembled those in healthy mice treated with physiological saline [75]. Furthermore, local injection of C2C12 myoblast-derived EVs into the injury site of bone-damaged mice significantly increased the bone-to-tissue volume ratio and accelerated bone recovery at the injured site [76]. Skeletal muscle-derived exosomes regulate bone metabolism by modulating the proliferation and function of BMSCs, osteoblasts, and osteoclasts.

Research has demonstrated that BMSCs can regulate muscle growth and development through exosomes [77]. For instance, exosomes derived from hypoxia-treated BMSCs deliver miR-210-3P to satellite cells in skeletal muscle, thereby modulating the expression of KLF7 and influencing the phosphorylation of the PI3K/AKT signaling pathway, which contributes to skeletal muscle regeneration [78]. Conversely, skeletal muscle can also influence the differentiation of BMSCs via exosomes and thereby affect bone metabolism [75]. Treatment of BMSCs with EVs isolated from normal mouse skeletal muscle reveals increased calcium deposition upon Alizarin Red staining compared to untreated controls. Additionally, the mRNA expression of osteogenic factors such as Runx2, Osterix, and Alp(Alkaline Phosphatase) is significantly upregulated. These findings indicate that EVs secreted by healthy skeletal muscle promote the osteogenic differentiation of BMSCs. However, in conditions such as muscle atrophy or other bone-related diseases, EVs secreted by skeletal muscle exert a negative regulatory effect on bone metabolism. Specifically, these EVs reduce calcium deposition during osteogenic differentiation of BMSC and inhibit bone formation [75].

The levels of ROS(reactive oxygen species) in skeletal muscle progressively increase with age. Upon stimulating C2C12 cells with hydrogen peroxide to mimic oxidative stress-induced aging and subsequently EVs, it was observed that the expression of miR-34a within these EVs was significantly upregulated. miR-34a is a microRNA closely associated with aging, and its expression in skeletal muscle increases with age. When EVs derived from hydrogen peroxide-stimulated C2C12 cells were used to treat BMSCs isolated from young mice, a marked reduction in BMSC number, decreased viability, and accelerated senescence were noted. Overexpression of miR-34a in C2C12 cells resulted in EVs that induced effects similar to those caused by oxidative stress stimulation. After being labeled and administered intravenously into mice, these exosomes were found to distribute predominantly in the forelimbs and hindlimbs. It is well established that miR-34a targets Sirt1 in BMSCs. Analysis of bone marrow cells extracted from these mice revealed downregulation of both Sirt1 mRNA and protein levels, contributing to senescence induction in bone marrow stem cells [79]. These findings suggest that during physiological aging, skeletal muscle can influence bone formation through alterations in miRNA expression within EVs, thereby accelerating bone loss and disrupting bone homeostasis. This insight provides a novel research direction for strategies to delay age-related bone loss.

Studies have demonstrated that diabetes can lead to reduced bone mass and density, as well as an increased risk of fractures [80]. In a diabetic mouse model induced by pancreatic β-cell toxin STZ, when bone defects occurred, local administration of C2C12 myoblast extracellular vesicles for nine days significantly accelerated the recovery of bone defects in diabetic mice. Further cellular studies revealed that exosomes released by C2C12 myoblasts could alleviate the suppression of mRNA expression of key osteogenic factors such as Osterix and Alp caused by AGE3(Advanced Glycation End-products 3). Additionally, these exosomes improved the delayed bone repair associated with diabetes by mitigating the reduction in osteocalcin expression at the injury site under diabetic conditions. The finding that exosomes reverse the negative effects of high glucose on osteogenesis at damaged sites provides a novel strategy for treating bone defects and fractures related to bone diseases. These results suggest that skeletal muscle-derived exosomes may serve as an effective tool for enhancing bone repair and regeneration.

Exosomes derived from skeletal muscle can transport LDHA(lactate dehydrogenase A) to BMSCs, thereby enhancing their glycolytic activity and promoting osteogenic differentiation. Furthermore, injecting exosomes isolated from healthy mouse skeletal muscle into BoNT/a-induced disuse osteoporotic mice significantly upregulates the expression of osteogenic markers such as Ocn(Osteocalcin) and Col-1 (Collagen Type I), improves the MAR(mineral apposition rate) in the distal femur, and mitigates bone loss caused by disuse osteoporosis. Notably, exercise increases the production of skeletal muscle-derived exosomes in mice, further amplifying these therapeutic effects [11, 81]. When exosomes secreted by C2C12 cells were labeled and applied to mouse embryonic osteoblast MC3T3–E1 cells, it was observed that the exosomes were internalized by the cells. This intervention increased Alp activity, enhanced matrix mineralization, and upregulated the expression of osteogenic factors such as Alp, Ocn, and Runx2, demonstrating that skeletal muscle-derived exosomes promote the osteogenic differentiation of MC3T3–E1 cells. Among these effects, the expression level of MiR-27a-3p was significantly elevated. miR-27a-3p activates the β-catenin signaling pathway by inhibiting APC(adenomatous polyposis coli) expression, thereby promoting osteogenic differentiation. Thus, miR-27a-3p plays a positive role in bone remodeling, and its inhibition suppresses osteogenic differentiation [82]. Importantly, skeletal muscle-derived exosomes not only regulate bone formation but also play a significant role in modulating bone resorption.

Under normal skeletal muscle function, exosomes secreted by skeletal muscle facilitate the osteogenic differentiation of BMSCs by transferring specific miRNAs and upregulating the expression of osteogenic factors, thereby enhancing bone formation [69]. An increasing body of research indicates that exosomes derived from different skeletal muscle cell subsets, such as satellite cells, myoblasts, and mature muscle fibers, exhibit significant heterogeneity in terms of cargo composition and functional output. Exosomes originating from satellite cells are rich in anti-fibrotic miRNAs, such as miR-206 and miR-133a. These exosomes not only support muscle regeneration but also indirectly maintain bone mass by slowing down muscle atrophy [67]. The exosomes secreted by mature muscle fibers mainly contain metabolic enzymes (such as LDHA) and osteogenic-related miRNAs (such as miR-27a-3p and miR-486-5p). These miRNAs directly promote osteoblast differentiation and mineralization by activating the β-catenin and mTOR signaling pathways [69, 82]. Moreover, FFSS-treated C2C12 myoblast-derived exosomes suppress osteoclast formation via the down-regulation of Nfatc1, and myoblast-derived exosomal miR-196a-5p and miR-155-5p may be involved in this process [83]. These findings also indicate that the functional specificity of extracellular vesicles derived from muscles is highly dependent on the developmental stage and physiological state of the secreting cells. And the key cargoes of extracellular vesicles from different muscle cell types and their effects on bone metabolism are also different.

**Fig. 3:**
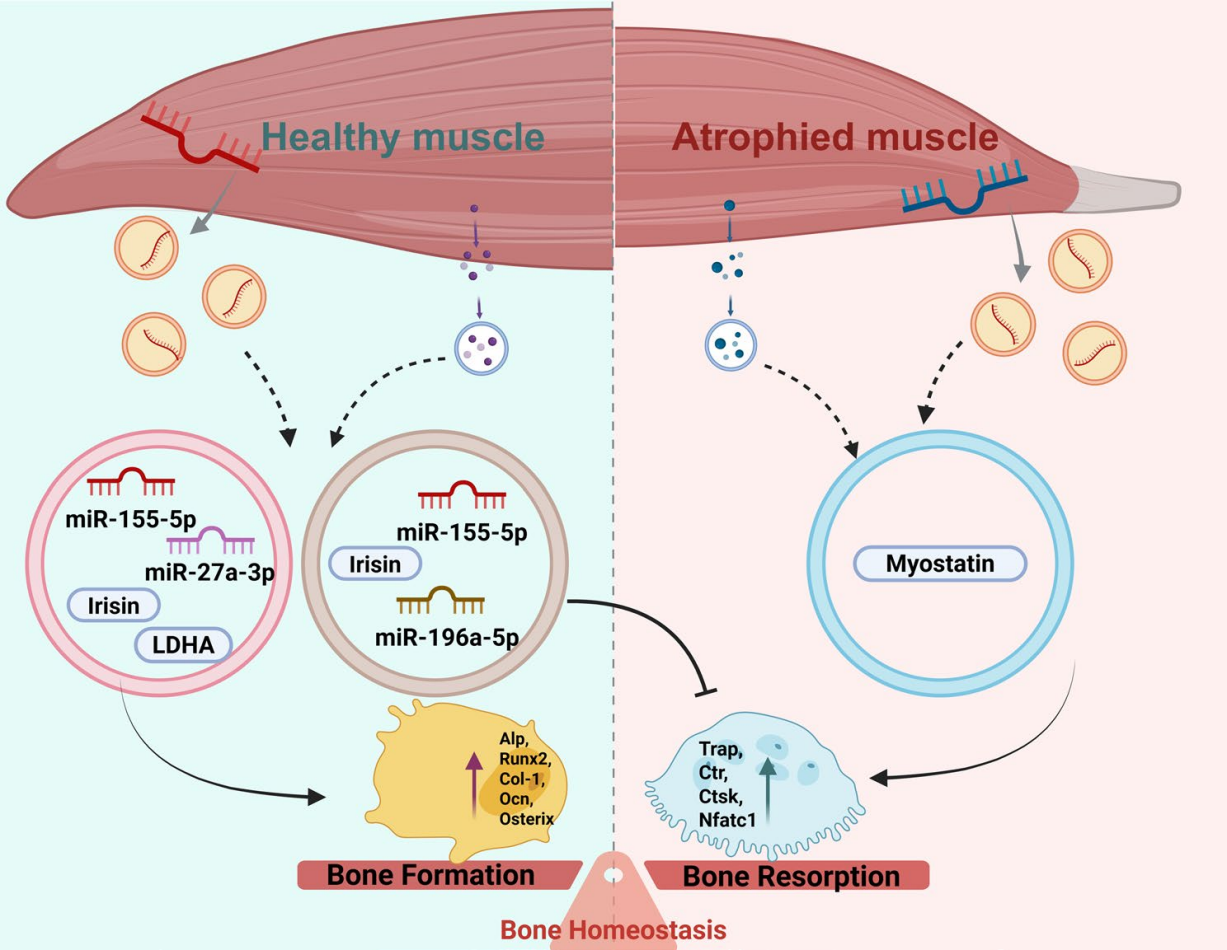
The impact of skeletal muscle-derived exosomes on bone homeostasis under various conditions. This figure elucidates the mechanisms by which exosomes contribute to bone formation and bone resorption under both healthy muscle and muscle atrophy conditions. In healthy muscles, Irisin facilitates bone formation via miR-27a-3p, thereby enhancing the expression of key bone formation-related proteins such as ALP, Runx2, Col-1, OCN, and Osterix. Conversely, under conditions of muscle atrophy, skeletal muscle-derived exosomes modulate myostatin or directly affect the expression of bone-resorption-related proteins, including TRAP, CIT, CTSK, and NFATc1. Overall, this figure highlights the pivotal role of exosomes in maintaining bone homeostasis

It’s worth mentioning that exercise can promote muscle function as well as skeletal muscle-derived exosomes (which promote osteogenesis) secretion [84]. Moreover, different exercise modalities exert differential regulation on both the secretory characteristics of exosomes and their subsequent effects on bone formation. For example, the combination of aerobic and resistance training significantly upregulates skeletal muscle mRNA levels of Clathrin and Alix—both involved in exosome biogenesis—thereby effectively enhancing skeletal muscle exosome secretion [85]. It has been reported that treadmill exercise exerts anti-osteoporotic effects by increasing irisin-loaded myogenic exosomes, which activate the AMPK–Nrf2 pathway in osteoblasts, promote osteoblast proliferation, and inhibit ferroptosis [86]. However, under conditions of abnormal muscle function or disease, skeletal muscle-derived exosomes may inhibit proliferation and osteogenic differentiation of BMSC by modulating miRNA expression, accelerating the senescence of bone marrow stem cells, and suppressing bone formation [66]. This bidirectional regulatory mechanism highlights the critical role of skeletal muscle-derived exosomes in bone metabolism, with their effects being contingent on the health status of the muscle (Fig. 3).

### Effects of skeletal muscle-derived exosomes on bone resorption

EVs derived from normal mouse skeletal muscle significantly inhibit osteoclast formation. When these EVs are added to an induction medium containing M-CSF(macrophage colony-stimulating factor) and RANKL(Receptor Activator of Nuclear Factor-κB Ligand), the mRNA expression levels of osteoclast-specific markers, including CTSK(Cathepsin K), Nfatc1, Trap, and Ctr(Calcitonin Receptor), are suppressed in osteoclasts derived from primary bone marrow cells. Trap staining further demonstrates that the number of Trap-positive osteoclasts is markedly reduced compared to the untreated group. Conversely, EVs derived from the skeletal muscle of amyotrophy-deficient mice exhibit the opposite effect, promoting osteoclastogenesis and disrupting the dynamic balance between bone formation and resorption, ultimately leading to bone loss [75]. These findings further suggest that the effects of skeletal muscle-derived EVs (exosomes) on bone metabolism depend on the functional state of skeletal muscle.

The EVs secreted by C2C12 myoblasts and EVs derived from skeletal muscle exhibit significant inhibitory effects on osteoclast differentiation in mouse bone marrow-derived cells, with similar intervention outcomes. As the number of EVs secreted by C2C12 myoblasts increases, the inhibition of osteoclast differentiation becomes progressively more pronounced. qPCR analysis demonstrated that the mRNA levels of key osteoclast-related markers, including Trap, Ctsk, and Nfatc1, were significantly reduced. It is known that the mitochondrial content of osteoclasts increases during their growth and differentiation [87]. Upon treating osteoclasts with EVs from C2C2 myoblasts, a marked decrease was observed in the mRNA levels of mitochondrial biogenesis markers such as PGC1β(Peroxisome Proliferator-Activated Receptor Gamma Coactivator 1β), ND4(NADH Dehydrogenase Subunit 4), and CytC(Cytochrome c). Notably, this treatment did not affect mitochondrial biogenesis in primary osteogenic cells isolated from mice. This effect occurs because EVs from C2C12 myoblasts can suppress the expression of ND4 and CytC, which are associated with the mitochondrial electron transport chain in Rankl-induced osteoclasts. These EVs prevent mitochondrial biogenesis by inhibiting mitochondrial metabolism during osteoclast formation.

Muscles and bones, as critical components of the musculoskeletal system, are significantly influenced by mechanical stress generated during exercise or physical activity (Table 1). This stress can modulate the levels of myogenic factors and signal transduction pathways within muscle cells. Upon applying FFSS(fluid flow shear stress) to C2C12 cells and subsequently collecting exosomes, no significant changes were observed in the number, distribution, or size of these extracellular vesicles. However, when FFSS-treated C2C12-derived exosomes were applied to mouse osteoclast precursors, they demonstrated a potent inhibitory effect on osteoclast formation. Compared with exosomes secreted by untreated C2C12 myoblasts, those subjected to FFSS intervention exhibited a markedly enhanced ability to suppress osteoclast differentiation. Further investigation revealed that FFSS-treated exosomes effectively downregulated the mRNA expression of key osteoclast-related factors, including Nfatc1 and DC-STAMP. Additionally, the expression levels of miR196a-5p and miR155-5p were significantly elevated in exosomes secreted by FFSS-treated C2C12 myoblasts [83]. Notably, miR196a-5p is a microRNA known to promote osteogenic differentiation while inhibiting osteoclastogenesis [88], whereas overexpression of miR-155-5p activates Wnt signaling, thereby enhancing osteogenesis and cell proliferation [89]. Collectively, these findings suggest that mechanical stress may regulate bone metabolism via miRNAs encapsulated in skeletal muscle-derived exosomes.

**Table 1:** Effects of skeletal muscle-derived exosomes on bone metabolism

Exosome Source	Study Models	Cargo	Target Tissues/Cells	Key Proteins/Enzymes	Effects	References
Skeletal muscle of Fndc5 transgenic mice	In vivo (transgenic mice)	Irisin↑	Osteoclasts	Irisin↑	Bone resorption↓	[72]
Healthy skeletal muscle of C57BL/6J mice	In vivo(Disuse osteoporotic mice)	-	BMSCs Osteoclasts	Runx2, Osterix, Alp↑	Bone formation↑ Bone resorption↓	[75]
C2C12 myoblast cell line	In vitro(H₂O₂ induces C2C12 cell senescence.)	miR-34a↑	BMSCs	SOD2↓, ROS-modified proteins↑	Bone formation↓	[79]
C2C12 myoblast cell line	In vitro	miR-27a-3p↑	Pre-osteoblasts MC3T3–E1	APC↓, Alp, Ocn, Runx2↑	Bone formation↑	[82]
C2C12 myoblast cell line	In vitro(pre-osteoclastic Raw264.7 cells)	-	Pre-osteoclastic RAW264.7 cells	Trap, Ctsk, Nfatc1↓	Bone resorption↓	[87]
C2C12 myoblast cell line	In vivo and in vitro(FFSS applied to C2C12 cells)	miR-196a-5p, miR-155-5p↑	Osteoclast precursors	Nfatc1, DC-STAMP↓	Bone resorption↓ Bone formation↑	[83]
Skeletal muscle of C57BL/6J mice	In vivo (Disuse osteoporotic mice)	LDHA↑	BMSCs	Ocn, Col-1↑	Bone formation↑	[11, 81]
Skeletal muscle of C57BL/6J mice	In vivo(Exercise intervention)	-	Osteoblasts	Irisin↑	Bone formation↑	[86]

This table lists some factors and miRNAs related to bone formation and bone resorption mentioned in this article, and also reveals the pathways through which they affect bone formation and bone resorption

**Fig. 4:**
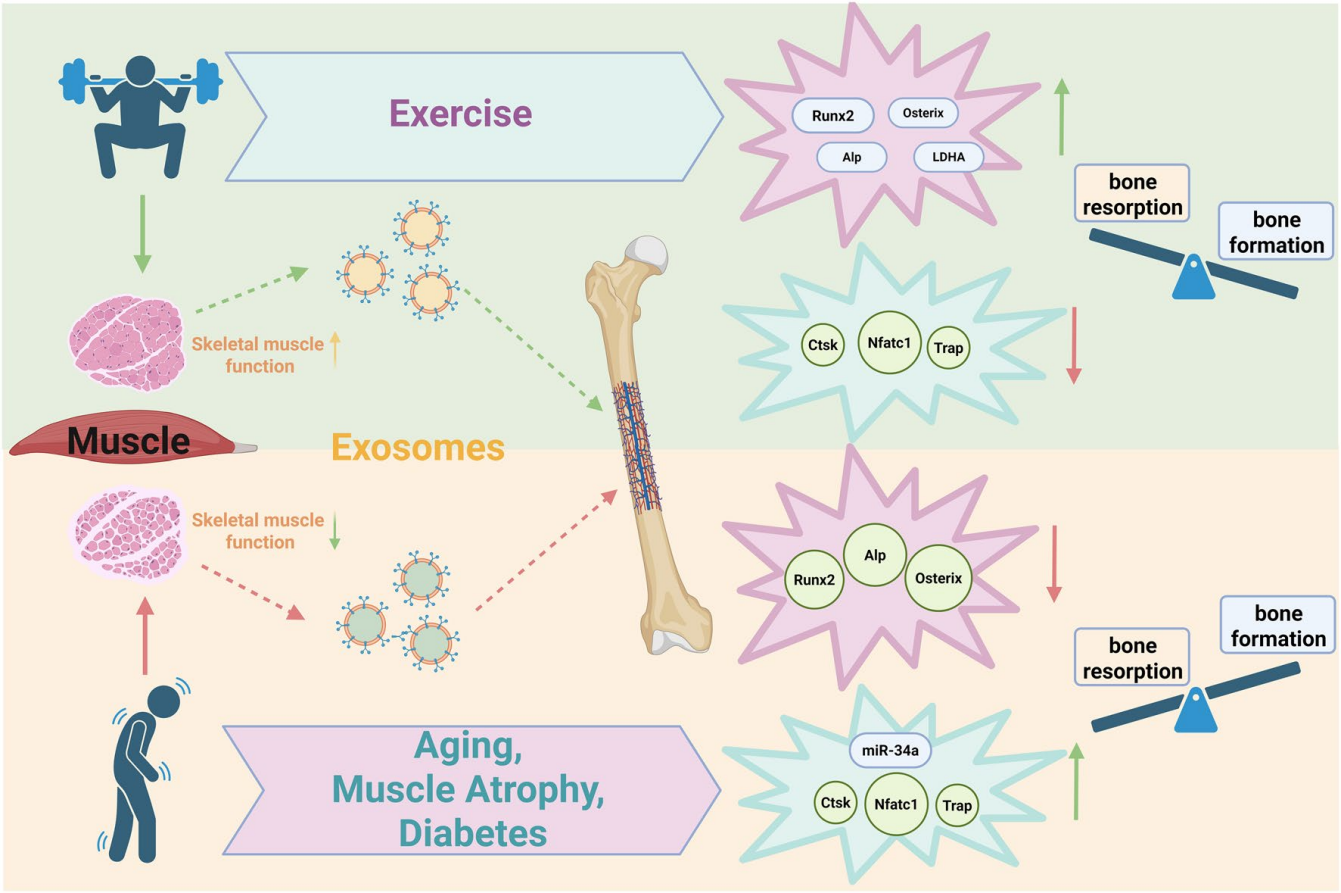
Effects of exercise and diseases on bone metabolism through skeletal muscle-derived exosomes. Exercise enhances skeletal muscle function, which releases exosomes that promote bone formation and inhibit bone resorption. In contrast, aging, muscle atrophy, and diabetes lead to decreased skeletal muscle function, resulting in the secretion of exosomes, which inhibit bone formation and promote bone resorption

Skeletal muscle-derived exosomes, which carry bioactive molecules such as miRNAs and proteins, play a critical regulatory role in both osteogenic and osteoclastic processes of bone metabolism. Research has demonstrated that exosomes secreted by skeletal muscle under normal or pathological conditions can actively mediate bone metabolism. Furthermore, mechanical stress can modulate the secretion of skeletal muscle-derived exosomes, thereby regulating bone metabolic processes. However, current in vivo studies investigating the effects of extracellular vesicles derived from skeletal muscle cells on bone following exercise intervention remain scarce. The underlying mechanisms of how exercise influences these processes warrant further exploration and clarification (Fig. 4).

Moreover, skeletal muscle-derived exosomes can also function as drug-delivery systems for bone-related diseases. For example, Skeletal muscle–derived exosomes, enriched in miR-27a-3p and miR-222-3p, markedly enhance the expression of osteogenic genes (Runx2, Osx, and Ocn) in BMSCs [9]. Upon exercise stimulation, skeletal muscle secretes exosomes enriched in miR-218, miR-27a-3p, and other microRNAs that travel through the bloodstream to bone tissue, enhancing osteoblast differentiation and bone formation [90]. This positions the exosomes as pivotal mediators of muscle–bone crosstalk and holds promise for targeted therapy of bone-related diseases.

## Summary

Exosomes are small vesicles secreted by most tissues in the body, containing bioactive molecules such as proteins, nucleic acids, and lipids. They can influence the functions of other cells and tissues via paracrine and endocrine pathways, playing a critical role in regulating bone metabolism. Skeletal muscle-derived exosomes exhibit high targeting specificity to bone tissue and hold promise as a potential therapeutic strategy for addressing bone-related diseases and facilitating drug delivery. Moreover, these exosomes can also modulate distant tissues through systemic circulation, indirectly influencing bone metabolism and offering novel insights into the mutual regulation between skeletal muscle and bone.

Skeletal muscles influence bone metabolism by secreting exosomes, which carry bioactive molecules such as miRNAs and proteins to modulate the balance between bone formation and resorption. Appropriate exercise can enhance the secretion of exosomes from skeletal muscles, thereby improving the therapeutic efficacy of osteoporosis. When skeletal muscle function is intact, the exosomes can deliver specific miRNAs (e.g, miR-27a-3p) and upregulate the expression of osteogenic factors, promoting bone formation. Conversely, exosomes secreted by skeletal muscles may suppress bone formation and exacerbate bone diseases in pathological conditions such as sarcopenia. Thus, exosomes serve as critical mediators in the crosstalk between skeletal muscles and bones.

At present, research on skeletal muscle-derived exosomes in the context of osteoporosis is gradually gaining attention. However, the mechanisms by which skeletal muscle-derived exosomes influence bone metabolism require further elucidation. Besides, additional in vivo experiments are warranted to comprehensively validate the impact of skeletal muscle-derived exosomes on bone metabolism.

With the development of biotechnology, it may be possible to develop exosomes capable of specifically targeting osteoporosis, thereby enhancing therapeutic efficacy while minimizing systemic side effects. Exercise has been demonstrated to regulate the secretion of skeletal muscle-derived exosomes. Future studies could further investigate the potential of skeletal muscle-derived exosomes to ameliorate osteoporosis under exercise intervention.

## Data Availability

Not applicable.

## Abbreviations

BMD: Bone Mineral Density
BMSCs: Bone Marrow Mesenchymal Stem Cells
CTSK: Cathepsin K
CTR: Calcitonin Receptor
DC-STAMP: DC-Specific Transmembrane Protein
FFSS: Fluid Flow Shear Stress
ILVs: Intraluminal Vesicles
IAC: Immune Affinity Capture
LDHA: Lactate Dehydrogenase A
MAR: microRNA
M-CSF: Macrophage Colony-Stimulating Factor
miR: microRNA
MVEs: Multivesicular Endosomes
NADH: Nicotinamide Adenine Dinucleotide
NLRP3: NOD-, LRR- and Pyrin Domain-Containing Protein 3
NTA: Nanoparticle Tracking Analysis
Ocn: Osteocalcin
Osterix: Osterix
PGC1β: Peroxisome Proliferator-Activated Receptor Gamma Coactivator 1β
RANKL: Receptor Activator of Nuclear Factor-κB Ligand
ROS: Reactive Oxygen Species
Runx2: Runt-Related Transcription Factor 2
Sirt1: Sirtuin 1
Trap: Tartrate-Resistant Acid Phosphatase
ULK1: Unc-51 Like Autophagy Activating Kinase 1
